# A protocol for a pragmatic randomized controlled trial using the Health Teams Advancing Patient Experience: Strengthening Quality (Health TAPESTRY) platform approach to promote person-focused primary healthcare for older adults

**DOI:** 10.1186/s13012-016-0407-5

**Published:** 2016-04-05

**Authors:** Lisa Dolovich, Doug Oliver, Larkin Lamarche, Gina Agarwal, Tracey Carr, David Chan, Laura Cleghorn, Lauren Griffith, Dena Javadi, Monika Kastner, Jennifer Longaphy, Dee Mangin, Alexandra Papaioannou, Jenny Ploeg, Parminder Raina, Julie Richardson, Cathy Risdon, P. Lina Santaguida, Sharon Straus, Lehana Thabane, Ruta Valaitis, David Price

**Affiliations:** 1 Department of Family Medicine, McMaster University, David Braley Health Sciences Centre, 100 Main Street West, 5th floor, Hamilton, ON L8P 1H6 Canada; 2 McMaster Family Health Team, Hamilton, Canada; 3 School of Nursing, McMaster University, Hamilton, Canada; 4 Clinical Epidemiology and Biostatistics, McMaster University, Hamilton, Canada; 5 Li Ka Shing Knowledge Institute, St. Michael’s Hospital, Toronto, Canada; 6 Department of Medicine, McMaster University, Hamilton, Canada; 7 School of Rehabilitation Science, McMaster University, Hamilton, Canada; 8 Institute of Health Management and Policy, University of Toronto, Toronto, Canada

**Keywords:** Primary healthcare, Older adults, Randomized controlled trial, Integrated care, Healthcare volunteers, Interdisciplinary healthcare teams, Implementation, Health services research, Personal health record

## Abstract

**Background:**

Healthcare systems are not well designed to help people maintain or improve their health. They are generally not person-focused or well-coordinated. The objective of this study is to evaluate the effectiveness of the Health Teams Advancing Patient Experience: Strengthening Quality (Health TAPESTRY) approach in older adults. The overarching hypothesis is that using the Health TAPESTRY approach to achieve better integration of the health and social care systems into a person’s life that centers on meeting a person’s health goals and needs will result in optimal aging.

**Methods/design:**

This is a 12-month delayed intervention pragmatic randomized controlled trial. The study will be performed in Hamilton, Ontario, Canada in the two-site McMaster Family Health Team. Participants will include 316 patients who are 70 years of age or older. Participants will be randomized to the Health TAPESTRY approach or control group. The Health TAPESTRY approach includes intentional, proactive conversations about a person’s life and health goals and health risks and then initiation of congruent tailored interventions that support achievement of those goals and addressing of risks through (1) trained volunteers visiting clients in their homes to serve as a link between the primary care team and the client; (2) the use of novel technology including a personal health record from the home to link directly with the primary healthcare team; and (3) improved processes for connections, system navigation, and care delivery among interprofessional primary care teams, community service providers, and informal caregivers. The primary outcome will be the goal attainment scaling score. Secondary outcomes include self-efficacy for managing chronic disease, quality of life, the participant perspective on their own aging, social support, access to health services, comprehensiveness of care, patient empowerment, patient-centeredness, caregiver strain, satisfaction with care, healthcare resource utilization, and cost-effectiveness. Implementation processes will also be evaluated. The main comparative analysis will take place at 6 months.

**Discussion:**

Evidence of the individual elements of the Health TAPESTRY platform has been shown in isolation in the previous research. However, this study will better understand how to best integrate them to maximize the system’s transformation of person-focused, primary care for older adults.

**Trial registration:**

ClinicalTrials.gov NCT02283723

**Electronic supplementary material:**

The online version of this article (doi:10.1186/s13012-016-0407-5) contains supplementary material, which is available to authorized users.

## Background

Healthcare systems are not well designed to help people maintain or improve their health. They are generally not well-coordinated and plagued with barriers to a person-focused care system [1, 2]. In Canada, current provincial healthcare systems are confronted with working to overcome disorganized connections among primary, secondary, and community care [3] and the use of processes that are reactive versus proactive [4]. Further, the current system is challenged by the burden of provider-patient interactions focused on a single disease rather than approaches that account for people who have multiple chronic conditions [4, 5]. Although the field of medicine has evolved over the years to focus on wellness versus illness, much change is still needed to take into account patient goals and preferences [6]. Finally, the system lacks coordinated strategies that address the social determinants of health [3, 7]. Transformational healthcare system change is needed if a person is to truly realize the World Health Organization’s (WHO) definition of health, “a resource for everyday life, not the objective of living; it is a positive concept, emphasizing social and personal resources, as well as physical capacities” [8].

Countries with a strong emphasis on primary healthcare have realized better health outcomes and health equity [9–11]. Effective primary healthcare is community-based, promotes healthy lifestyles as a pathway to disease prevention, provides ongoing care for chronic conditions, and recognizes the importance of the broad determinants of health [12]. Primary healthcare embraces a wide suite of services and involves a broad range of healthcare providers in a manner that is person-focused and coordinated [13]. It has been identified that integrated care allows a person to plan their care with people who work together to understand them and their caregivers, allow them control, and bring together services to achieve outcomes of importance to them [14]. A strong primary healthcare system also needs to be well integrated with the rest of the healthcare system. The WHO defines integrated care as the bringing together of inputs, delivery, management, and organization of services related to diagnosis, treatment, care, rehabilitation, and health promotion [15]. High-quality integration encompasses better care delivery horizontally through improved interdisciplinary team collaboration and vertically across different levels of care such as in the home, primary, secondary, and tertiary care [15]. Taking into account complexity theory concepts when considering effective healthcare delivery also recognizes that delivery of healthcare itself is a complex adaptive system and, as such, changes according to current demands [16, 17]. Therefore, improving primary healthcare requires the many components of the healthcare system to undergo whole-scale transformational system change and to do so in a manner that produces person-focused coordinated care.

Individuals ≥65 years are the fastest growing age group in Canada. This age segment in July 2015, for the first time ever, outnumbered people aged ≤15 years [18-21]. Worldwide, the number of people over the age of 60 is expected to double by 2050 [19, 20]. If no improvements are made to the current healthcare system, greater numbers of people will have unmet healthcare needs that will hinder healthy aging [20, 21]. Many older adults report being in excellent or very good health and are able to carry on daily activities on their own [22]; however, it is also well recognized that age is associated with increased numbers of chronic conditions [23], which can lead to functional decline and increased use of the healthcare system [19, 23, 24]. A well-functioning healthcare system will help keep people healthy and living at home and properly supported. Further, a person-focused approach has been proposed to tackle multimorbidity [25, 26].

### Theory and development approaches

The chronic care model was used to identify key themes and players that should be incorporated into the intervention [27]. Six core elements of the chronic care model were incorporated: healthcare organization and leadership, linkage to community resources, support of client self-management, coordinated delivery system design, clinical decision support, and clinical information systems [27] (see Table 1). A five-pronged approach has been used to develop the Health Teams Advancing Patient Experience: Strengthening Quality (Health TAPESTRY) platform: (1) developmental evaluation [28, 29], (2) participatory co-development [30, 31], (3) formal investigation of sustainability [32], (4) iterative pilot testing [30] including evaluation of implementation of the intervention, and (5) a pragmatic randomized controlled trial (RCT) to evaluate the effectiveness and cost-effectiveness. The combination of approaches allows the team to explicitly record and gain progressive insight into key decisions made during development including those intended to foster sustainability and scalability, ensure the views of key players were incorporated, and allow for improvements to the intervention to be made and tested in an iterative manner before large scale evaluation.

**Table 1:** Chronic care model and health TAPESTRY

Element	Definition	Health TAPESTRY
Healthcare organization and leadership	Strong leadership, readiness for change, and effective incentives to systematically promote successful quality improvement interventions.	Health TAPESTRY creates time and space for clinic huddles to take place to discuss clients individually in an organically defined, interprofessional process through support from clinic leadership.
		Clinic leadership supports integration of volunteers into the team and the adoption and use of e-health technologies
Linkage to community resources	Efficient use of community resources such as peer-support groups, community programs, and counselling to improve the quality of care and support offered to patients and improve cost-effectiveness in the system.	Health TAPESTRY offers linkages to community organizations through support of volunteers and directed healthcare provider referral or connection based on goals and needs oriented action plans.
Support of patient self-management	Patient empowerment, activation, and support of self-management skills to effectively sustain management of chronic conditions.	Health TAPESTRY volunteers serve as advocates for clients and encourage self-management through follow-up and discussion of client-identified health goals. Healthcare providers encourage self-management activities through education and actions based on goals and needs oriented action plans.
Coordinated delivery system design	Disconnected care across multiple providers and caregivers is a point of inefficiency in the health system; therefore, addressing lack of coordination to significantly improve patient experience.	The KindredPHR seeks to allow clients to better connect to all their providers in a more coordinated way. The Health TAPESTRY specific applications generate information in the home that is shared electronically with the clinic.
Clinical decision support	Facilitating the use of evidence-based guidelines and patient assessment tools to enhance effectiveness.	The Health TAPESTRY App contains modules (surveys, risk algorithms) that have been supported by evidence and expert opinion.
Clinical information systems	Improving patient-provider and provider-provider communication, using reminder systems, documenting treatment plans and sending secure messages to enhance the delivery of proactive care.	The KindredPHR offers secure messaging between clients and providers and allows for establishment of reminders, tracking of health information and treatment plans, and recording data. Communication is also be facilitated by the clinic EMR.

*PHR* personal health record, *EMR* electronic medical record

### Evidence supporting the elements of Health TAPESTRY

Health TAPESTRY seeks to bring together elements of healthcare delivery into a combined platform that capitalizes on current system strengths yet moves these forward using an integrated healthcare approach. Specifically, Health TAPESTRY centers on meeting a person’s health goals with the support of trained community volunteers, technology, an interprofessional team, system navigation, and community engagement. Healthcare volunteers have been shown to provide social support that is both physically and emotionally therapeutic to patients [33, 34]. Secondly, a multitude of benefits have been report from electronic medical records (EMRs) [35–37]. Additionally, with some mixed evidence, patient on-line access to their own health information (through personal health records (PHR)) has been shown to improve patient self-care, health outcomes, and communication and engagement with clinicians [38, 39]. Thirdly, much evidence supports the positive impact of team-based care on numerous health outcomes across several chronic diseases [40–42]. Finally, system navigation has been associated with important health outcomes [43, 44] and helps to address social determinants of health [45, 46].

We are unaware of any studies that integrate the components of Health TAPESTRY into a coordinated approach intended to improve delivery of primary healthcare. In essence, Health TAPESTRY is a health and social care approach that centers on meeting a person’s health goals and health needs explicitly gathered with the support of technology, community volunteers, an interprofessional team, and system navigation and better links between primary care and community organizations. It is a complex, multilevel approach to integration, from both system level and individual level perspectives.

The overarching aim of the Health TAPESTRY platform is to promote optimal aging. Health TAPESTRY intends to promote optimal aging through (1) intentional, proactive conversations about a person’s life and health goals and health risks and then initiation of an action plan that supports achievement of those goals and addressing of health risks; (2) improved collaborative working within the interprofessional primary care team, community service providers, and informal caregivers; (3) training volunteers to serve as a link between the person in their home and their primary care team; and (4) using technology including the PHR that allows personal health information and patient health goals to link directly to the EMR with the primary healthcare team.

### Overall objective, research questions, and hypothesis

We aim to evaluate the implementation and effectiveness of the Health TAPESTRY approach in older adults. The primary research question is, what is the effectiveness of the Health TAPESTRY approach on the identification and attainment of a person’s health goals in older adult participants compared to people not receiving the Health TAPESTRY approach? Our hypothesis is that better integration of the health and social care systems into a person’s life will allow a person to better attain their health goals. Four secondary research questions will also be asked with respect to several participant outcomes, cost-effectiveness, sub-analyses, and intervention duration (see Additional file 1 for a complete list of secondary research questions). An additional set of questions will be asked related to the processes of implementation of each component of the Health TAPESTRY approach (see Additional file 1).

## Methods/design

### Design

**Fig. 1:**
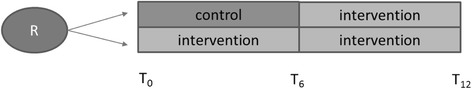
Study design. *R* randomization, *T*_*0*_ baseline, *T*_*6*_ 6-month time point, *T*_*12*_ 12-month time point

This is an unblinded delayed intervention pragmatic RCT. Participants in the control group will receive the intervention at 6 months, and both groups will continue to be part of the study until the 12-month mark (Fig. 1). The main analysis will be group comparisons at 6 months. Both groups will be also assessed at 12 months. Ethics approval was granted from the Hamilton Integrated Research Ethics Board (8 December 2014). The trial has been reregistered with Clinical Trials.gov NCT02283723. We used the SPIRIT guidelines to guide reporting of our trial protocol [47].

### Trial setting

The study will be performed in Hamilton, Ontario, Canada, at the McMaster Family Health Team (MFHT) and surrounding communities. The MFHT consists of groups of family physicians and other healthcare professionals, providing 7-day-a-week access to care, supported by an EMR, and providing a broad collection of services based on community needs. The MFHT is paid using a blended model of funding, including capitation and fee for services, bonuses for achieving prevention targets, and special payments to expand the scope of care [48]. The MFHT has approximately 32,000 rostered patients, 36 family physicians, 74 medical residents, 15 locums, 18 nurses, and 26 allied healthcare professionals.

### Study participants

As a planned pragmatic RCT, efforts will be made to limit the restrictiveness of the trial inclusion criteria. Inclusion criteria included patients rostered with MFHT, aged 70 years or older, and living in Hamilton, Ancaster, Dundas, Stoney Creek, Grimsby, Caledonia, or Rockton, Ontario. Exclusion criteria included people who reside in long-term care, will be out of the country for more than 50 % of trial duration, are palliative or receiving end-of-life care, or do not speak English or have a family member who speaks English. An initial list of potential participants will be generated through a query using the clinical EMR. Family physicians will screen lists of possible participants for exclusion criteria, and then invitation letters will be sent to all remaining participants. All volunteers and healthcare team members who are involved in the study will be invited to participate to provide their perspective as part of the implementation evaluation.

### Randomization and blinding

While it is recognized that there could be potential contamination between intervention and control groups by the primary care providers who will have participants in both arms, the intervention focuses on identifying a person’s health goals which a salient component to all other actions to take place as part of the intervention. Thus, without the processes in place to receive information about a person’s goals, contamination is unlikely, and so it was felt that clustering at the level of the practice was not necessary.

The randomization process will involve an automated central (allocation concealed) computerized randomization sequence, with the patient as the unit of randomization. Randomization will be stratified by participant gender and MFHT site (McMaster Family Practice or Stonechurch Family Health Centre). Couples will be accepted into the study to encourage study participation and to ensure equitable access to the intervention. Recognizing the potential influence of one member of a couple on the other, both members will be randomized into the same group. One member of the couple will be randomly selected to contribute their data to the main analysis.

Participants, caregivers, and volunteers will not be blinded; however, there will be some masking of groups: participants will not explicitly be told that they are in the initial or delayed group. Physicians and other members of the healthcare team will be not be formally blinded to allocation; however, there will be some masking as they will only know a participant is receiving the intervention once a Health TAPESTRY report is reviewed and may not recognize whether a person has been allocated to the intervention or control group. Data analysts will be blinded to the study group.

### Intervention

**Fig. 2:**
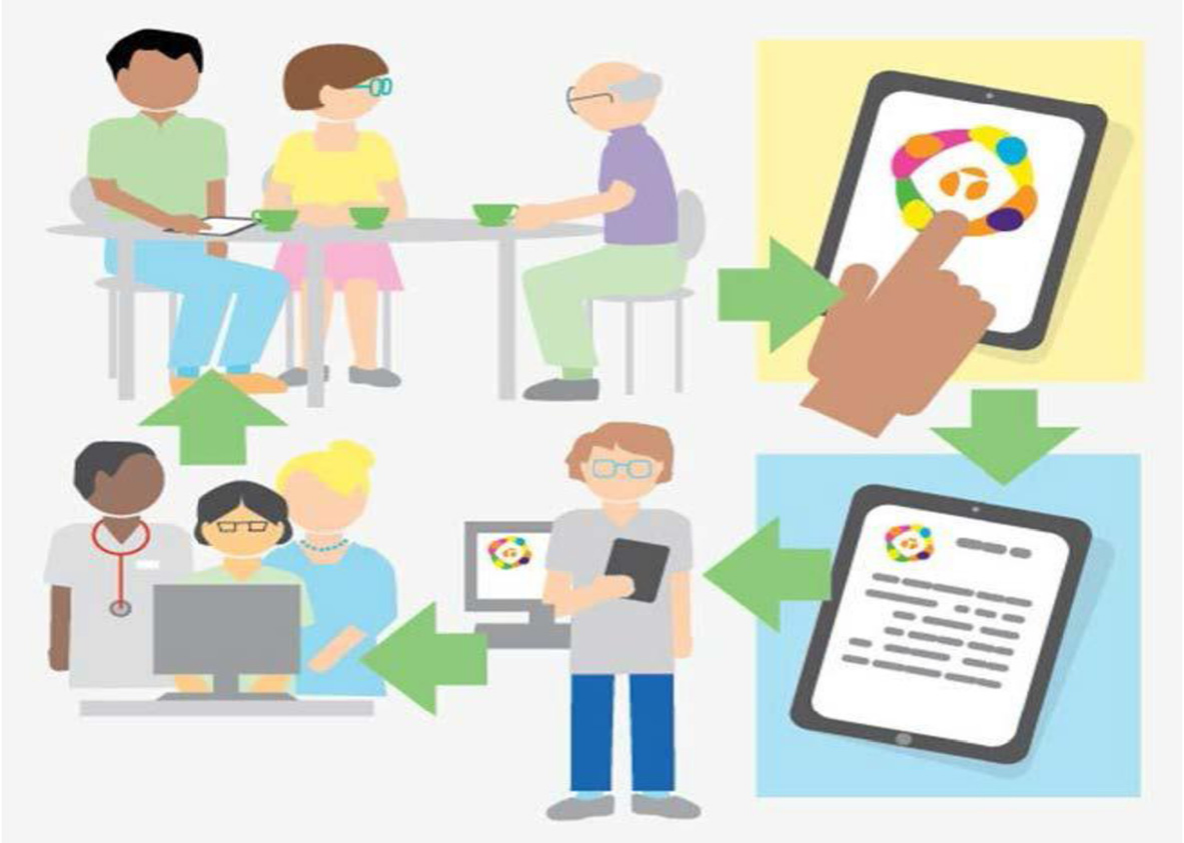
Health TAPESTRY process. Volunteers visit clients in their home and use the Health TAP-App to collect information. This information is summarized on a Health TAPESTRY report and is uploaded into the person’s electronic medical record to be shared with the intake team at the clinic. Reports are viewed and an action plan is developed which can include community organizations and resources, volunteer follow-up visit, and follow-up in any nature by healthcare team members and the client using their personal health record. The Health TAPESTRY process continues in an iterative fashion until the participant is discharged from the program

A visual depiction of the study flow and planned timeline is provided in Additional file 2. A detailed description of the intervention is provided in Additional file 3. A screenshot of the virtual learning centre and training modules are shown in Additional File 4. A brief summary of the intervention has been described and is visually depicted in Fig. 2. A client (the research participant) will receive a visit in their home from a pair of trained volunteers. The volunteer pair will collect information electronically using a tablet computer which houses a specifically designed Health TAPESTRY software application (TAP-App). Information such as life and health goals, daily life activities, and general health will be collected using structured surveys and unstructured narratives (see Additional file 3). The information gathered will be organized in a report summarizing any alerts, key issues, observations, and goals. This report will be securely sent electronically to an intake team at the clinic. Intake teams may include any combination of healthcare professionals and were put in place to address high-health-need patients including high users of the healthcare system and Health TAPESTRY clients.

Each clinic will decide which healthcare team members should be included in the intake team and define a process that works best within their workflow. The clinic intake teams will review the reports and connect with the appropriate interprofessional healthcare team across the MFHT making sure to include the most responsible family physician. Collectively, a care plan is generated to facilitate how the team, community agencies, and the volunteers can address the client’s goals and health issues. The intervention treatment period will be 6 months.

### Control

The control group will receive the usual care. These individuals will not have volunteer visits or be discussed at an intake meeting. They may or may not have had a previous PHR account. At the conclusion of the 6-month trial, the control group will have the option of receiving the intervention.

### Follow-up

Follow-up for the purposes of research will be according to intention to treat (ITT) and take place throughout implementation (qualitative data collection) at 6 and 12 months (quantitative data collection) in the intervention and control groups. Clinical follow-up during the intervention will be determined by clinical needs.

### Outcomes

Tables 2 and 3 summarize the measures, timing, and data collection method used for the primary and secondary outcomes, in addition to the measures and timing of the implementation processes. The following section gives more detail for clarity and is broken down into two broad sub-sections: client outcome measures and implementation processes. A description of the tracking of adverse events and the economic analysis is also included.

**Table 2:** Summary of trial client outcomes and implementation processes

Client outcomes				
Variable/outcome	Hypothesis	Outcome measure	Timing	Methods of analysis^a^
Goal attainment	GAS score will be higher in the intervention arm compared to the control arm at 6-months; proportion of participants who report maintaining or improving in the top priority goal area will be higher in the intervention arm than the control arm at 6-months	Goal attainment scaling score [53] obtained from a structured interview; proportion of participants who report maintaining or improving in their top priority goal area	*T*_6_, *T*_12_	Linear regression for continuous variables; logistical regression for categorical variables
Self-efficacy	*Higher* self-efficacy, quality of life, optimal aging, social support, access, comprehensiveness, patient empowerment, patient-centeredness, and satisfaction of care and *lower* caregiver strain, hospital admissions, and emergency room visits will be reported in the intervention arm compared to the control arm at 6-months	Self-efficacy for managing chronic disease [55]; self-report	*T*_0_, *T*_6_, *T*_12_	
Quality of life		EQ5D-5L [56]; self-report	*T*_0_, *T*_6_, *T*_12_	
Optimal aging		Single-item optimal aging question from the Canadian Longitudinal Study on Aging [57]; self-report	*T*_0_, *T*_6_, *T*_12_	
Social support		DUKE social support index [58]; self-report	*T*_0_, *T*_6_, *T*_12_	
Access		CIHI common indicators; self-report [59]	*T*_0_, *T*_6_, *T*_12_	
Comprehensiveness of care			*T*_0_, *T*_6_, *T*_12_	
Patient empowerment			*T*_0_, *T*_6_, *T*_12_	
Patient-centeredness			*T*_0_, *T*_6_, *T*_12_	
Caregiver strain		4-item Zarit screen [60]; self-report	*T*_0_, *T*_6_, *T*_12_	
Satisfaction with care		Single item, satisfaction rated from 1 to 10; self-report	*T*_0_, *T*_6_, *T*_12_	
Hospitalizations and emergency room visits		EMR abstraction	*T*_0_, *T*_6_, *T*_12_	

Demographic information including age, gender, level of education, marital status, ethnicity, language spoken, and number of medications, falls, adverse events, and economic analysis not included in the above table

*T*_*0*_ baseline, *T*_*3*_ 3-month collection, *T*_*6*_ 6-month collection, *T*_*12*_ 12-month collection

^a^Analyses will be adjusted for participant gender and MFHT site (McMaster Family Practice or Stonechurch Family Health Centre)

**Table 3:** Summary of trial client outcomes and implementation processes

Implementation processes			
Variable/outcome	Outcome measure	Timing	Methods of analysis
Volunteer self-efficacy	3-item self-efficacy rating of communication with client, performance of tasks necessary for home visit (i.e., navigate TAP-App) and handling unexpected issues (0–100 % scale)	Prior to each home visit	Mean differences for the first 3 months of visits compared to the second 3 months of visits
Completeness of TAP-App used by volunteers	Time to complete each module; number of items missed	*T*_6_, *T*_12_	Mean differences where applicable
Client satisfaction with volunteers	Description of experience with volunteer home visits	*T*_3_, *T*_12_	Qualitative descriptive method
Uptake of personal health record by clients	PHR metrics including number of times logged in, features used, number of secure messages sent, issues encountered, and described experience with the personal health record	*T*_0_, *T*_6_, *T*_12_	Mean differences where applicable; content analysis with frequency counts of each category where applicable
Type and extent of healthcare team involvement with client	Chart audit to track actions of healthcare team (type of follow-up, team members involved)	*T*_6_	Content analysis with frequency counts of each category of resource
Quality and extent of healthcare team functioning and organizational readiness for change by health team members	Described in qualitative interviews	*T*_3_, *T*_12_	Qualitative descriptive method
	ORCA [66]		Mean difference between *T*_3_ and *T*_12_
How often and what types of community resources are utilized by clients?	Chart audit to track connections to community resources and programs	*T*_6_	Content analysis with frequency counts of each category of resource
Type and extent of involvement of clients in program	Described in qualitative interviews	*T*_6_	Qualitative descriptive method
Type and extent of involvement of family caregivers in the program	Research session notes; described in qualitative interviews	*T*_6_	Content analysis with frequency counts of each category of resource
What life and health goals are generated by clients?	Structured open-ended questions in research session	*T*_6_, *T*_12_	Thematic analysis of goals based on goal areas
Process of implementation and factors influencing implementation	Described in qualitative interviews	*T*_3_, *T*_12_	Qualitative descriptive method

Demographic information including age, gender, level of education, marital status, ethnicity, language spoken, and number of medications, falls, adverse events, and economic analysis not included in the above table

*T*_*0*_ baseline, *T*_*3*_ 3-month collection, *T*_*6*_ 6-month collection, *T*_*12*_ 12-month collection

#### Client outcome measures

Data on client demographic and other characteristics, as well as outcome measures, will be gathered using multiple data collection methods including self-report client surveys, clinic EMR, volunteer-administered TAP-App, and research program records. Outcome data measures will be repeated at 6 and 12 months. Client surveys will be programmed into the software administered by the volunteers during home visits or into software (REDCap, Version 6.9.7, Vanderbilt University [49]) used by researchers.

Data abstraction from clinic EMRs will be carried out using a structured, pilot-tested form. Chart audit of each patient examining the number of visits, the clinicians involved, and any referral or consultations internally or externally, as well as any physical measurements such as blood pressure, fasting blood glucose level, and lipids will take place at the end of the study. Data abstraction will occur independently in duplicate until reasonable agreement has occurred (i.e., 0.70), as calculated by Kappa statistic [50, 51]. Administrative data on health services utilization will be acquired from the EMR.

#### Primary outcome

The primary outcome is the mean difference in the goal attainment scale (GAS) score at 6 months over baseline in the intervention arm, compared with the control group [52]. Participants will identify health goals, indicators, and outcomes through structured, prompted discussion. Each participant will rate their progress towards achievement of their goal using a set of five expected outcome levels for each indicator related to that of health goal. Outcome levels identified will be as descriptive, objective, and observable as possible and phrased as relative versus absolute improvements [53]. At 6 months, a GAS score will be calculated for each participant according to conventional methods [52] and used as the primary outcome. The proportion of participants’ self-reporting maintenance or improvement in their top priority goal area will be used as a secondary outcome. Further details about the process for gathering data to create the GAS are provided in Additional file 5.

#### Secondary outcomes

A number of secondary outcomes will be collected (see Tables 2 and 3 for the description and reference of each outcome). Self-efficacy for managing chronic disease [54], quality of life (EQ-5D-5L) [55], optimal aging [56], social support [57], caregiver strain [58], and satisfaction with care will be measured. Also, access to healthcare services received at their clinic, comprehensiveness healthcare services received at their clinic, patient empowerment, and patient-centeredness will be measured using standardized questions developed by the Canadian Institute of Health Information through the Canadian Institutes of Health Research Community-Based Primary Healthcare initiative [59]. Caregiver strain will be measured using the four-item Zarit screen [58]. Hospitalizations and emergency room visits will be categorized into (1) those related to ambulatory care sensitive conditions for chronic disease [60, 61], (2) those related to adverse effects, and (3) overall for any reason.

#### Implementation processes

Qualitative data will be analyzed to help understand the implementation of the intervention including the type and extent of involvement that participants, family caregivers, volunteers, and healthcare team members have in this program, volunteer confidence, and factors influencing implementation of the intervention from the perspectives of all types of participants including factors to consider when adapting the Health TAPESTRY approach to other contexts.

Specifically, the focus group and interview guides for the use with healthcare provider team, volunteers, and client participants will consist of pilot-tested open-ended questions that will address the structures and processes of care, perceived barriers and facilitators of implementing and the use of all components of the intervention, and what worked and did not work in implementing the program and in using each component. The focus group and interview guides are based on normalization process theory [62, 63]. Modifications to the interview guide will occur as themes emerged. Weekly field notes taken during primary healthcare intake team meetings will add rich detail to track the process of implementation of the intervention and any changes made over time. Data will be collected at 3 and 12 months of the intervention. In addition, to maximize Health TAPESTRY’s potential for impact, we assessed the intervention’s sustainability and scalability potential prior to implementation in this current trial using a mixed-methods approach [32]. This work involves (1) administering a sustainability survey (a validated instrument developed by the National Health Service (NHS) institute [64]) to team members who were involved in the intervention development and pilot testing (*n* = 20) and (2) a qualitative study of one-on-one telephone interviews with a subset of NHS survey respondents (*n* = 25) to gain a more in-depth understanding of the factors that influence the implementation, sustainability, and scalability of the Health TAPESTRY intervention. Findings from these studies were used to optimize the intervention in preparation for the current trial [32].

To compliment the qualitative data collected and to further understand the implementation of the intervention and the readiness of healthcare team members for change, quantitative data will also be collected. Organizational readiness of members across MFHT will be measured using the Organizational Readiness for Change Assessment (ORCA) [65]. Time between volunteer completion of data collection, report placement into the EMR, and discussion by intake team will also be recorded and tracked across the trial. The follow-up actions of the healthcare team will be described via chart audit of the EMR. Action type including communication method and the actions made by the healthcare team will be examined. Types of community resources recommended and utilized by participants will also be recorded. Finally, the types of life and health goals generated by participants and the proportion of recommendations made or actions taken to address these goals will be recorded. Other implementation elements complimented by a quantitative measure are volunteers’ self-efficacy to fulfill their role as a volunteer, the completeness of the initial assessments completed by the volunteers during home visits, and PHR uptake and use (see Tables 2 and 3 for descriptions).

#### Adverse events

Details of adverse events or harms from any source will be reported to the research team and recorded on a structured form. Follow-up will be completed by the appropriate person and documented. All critical incidents identified by volunteers, including potential adverse events, will be reported and followed up using a standard protocol.

#### Cost-effectiveness

Incremental costs and effects (utility) will be calculated and if the Health TAPESTRY program is both more costly and more effective, an incremental cost-utility ratio will be calculated showing how much more it costs for a QALY gained, using EQ-5D-5L [55] as the indicator of quality of life. If the Health TAPESTRY program is equally effective as no Health TAPESTRY program, then the economic evaluation will be based solely on incremental costs (see Additional file 6 for full details).

### Data analysis

Tables 2 and 3 include a summary of methods of data analysis for each variable. We have provided more details related to the statistical data analysis plan for quantitative data related to client outcomes, in addition to describing the economic data analysis and data analysis for the qualitative data related to implementation processes.

#### Client outcomes

We propose to test using two analysis sets: ITT set, considering all patients as randomized regardless of whether they received the intervention and a “per protocol” analysis set. A per protocol analysis will include all participants who received the intervention including both members of a couple assigned to the intervention group compared with all participants randomized to the control group including both members of a couple assigned to the control group.

Quantitative data analysis will include descriptive analyses with means and standard deviations calculated for the continuous variables and frequencies calculated for categorical variables. Data will be summarized in tabular or graphical form. The main between-group comparison will take place at 6 months. This point also serves as the control group’s baseline as they move into the intervention arm based on the delayed design. The comparison at 12 months then compares the effect of the intervention after 12 months of participation (original intervention group) versus 6 months of intervention (original control group).

The analysis will be blind to study group allocation and will follow ITT principle. Dropouts will not be replaced. Reporting will follow the CONSORT extension for pragmatic randomization trials [66] and non-pharmacological interventions [67]. The baseline characteristics of the practices and patients will be reported by group as mean (standard deviation) or median (first quartile, third quartile) for continuous variables, depending on the distribution, and count (percent) for categorical variables. Multiple imputations will be used to handle missing data to enable ITT analysis. The data will be analyzed using generalized estimating equations—assuming exchangeable correlation structure adjusting for baseline scores, to analyze all outcomes [68]. The results will be reported as estimate of the effect, corresponding 95 % confidence interval and associated *p* values. All *p* values will be reported to three decimal places with those less than 0.001 reported as *p* < 0.001. The criterion for statistical significance will be set a priori at alpha = 0.05 and adjusted using the Bonferroni method for multiple secondary analyses. Analyses will be performed using SAS V9.4 (Cary, NC). There will be a single final analysis at the end of the trial. Subgroup analyses will include examining differences between the following groups: men and women; age less than 80 and 80+ years; those residing alone and residing with others; or those with three or more chronic conditions.

#### Econosmic analysis

The economic analyses will include program cost measures, health resource use and cost, and patient quality of life. The cost-effectiveness/utility analysis will be conducted from the Ontario government healthcare system perspective.

#### Implementation processes

For quantitative indicators (volunteer self-efficacy, completeness of the TAP-App, PHR uptake, and the ORCA), a paired *t* test will be conducted to examine the mean differences between relevant time points. A descriptive method [69] will be used to explore implementation questions collected via qualitative methods. An inductive and deductive analysis approach will be used. Using NVivo 10 software, open coding will be used to capture participants’ perspectives of implementation and to track the process of implementation from field notes. These codes will be collapsed under pattern codes [70] which will be organized under normalization process theory constructs to track factors influencing implementation. An initial code book will be derived from the first two to three discussion groups and revised as needed to capture emerging themes. Two research team members will review all transcripts and notes pages and develop a codebook to capture emerging themes. Analysis will focus on implementation and understanding how each key group fits within Health TAPESTRY as well as their needs within the intervention. NVivo 10 matrix queries will be used to examine differences and similarities in perceptions based on of each group (healthcare provider, volunteer, and client). Data collection will take place concurrently with data analysis, so as to ensure that new themes are fully explored.

#### Power and sample size

Assuming baseline scores of 30 on the GAS score in intervention and control groups and a standard deviation of 15 with a power of 80 % and type I error probability of 0.05, we will need to enroll 286 patients overall to detect a mean difference of 5 points on the GAS score representing improvement in the level of goals attained in the intervention group compared with control [71–73]. Previous studies in older adults have demonstrated that this difference indicates a meaningful change in effect and is achievable [71–73]. This sample size will also give sufficient power to detect a difference of 1.0 (SD, 2.5) on the self-efficacy for managing chronic disease scale [54] and a difference of 0.10 (SD, 0.3) on the EQ-5D-5L measure, both identified as meaningful differences. The study will not be powered for planned subgroup analyses, and so these will be considered exploratory. To account for a 10 % potential loss to follow-up in the study, we will aim to enroll 316 participants.

### Trial status

This trial is in the study recruitment phase. We expect the final 6-month follow-up period for intervention and control participants to occur in April 2016.

## Discussion

The Health TAPESTRY platform has set laudable goals. It aims to remodel community-based primary healthcare to better reach people in their homes to intervene ahead of critical need. It aims to use accessible technology and a network of volunteers, to let clients self-direct and monitor their care, and make better connections to their healthcare team. It aims to break down barriers between people in their homes and community partners and healthcare teams. Any change in healthcare delivery necessitates rigorous evaluation to understand whether new processes generate the desired improvements. Multiple pilot tests have studied various components of the Health TAPESTRY platform to demonstrate feasibility and process improvements. This protocol describes the plans to rigorously evaluate the Health TAPESTRY community-based primary healthcare platform to understand whether it creates a positive difference in the achievement of their individual health goals or in how people experience the healthcare system.

The study uses the GAS score as a primary outcome. The strength of the GAS process is that it applies well to the situations of multimorbidity because it inherently allows the outcome to be individualized to the patient’s priority areas across their personal set of multimorbidities. It is also a person-focused measure because it only measures areas applicable to that particular person. The GAS process enables a patient and healthcare provider to work together to identify the patient’s priority problem areas and to systematically set realistic goals around that particular problem area. It allows for the negotiation of realistic, observable, and objective goals that could be attained with changes in health management, and it measures whether goals have been met at pre-specified follow-up times [74, 75]. The process is congruent with individualized clinical care but also provides a framework to consider as a measurement approach because it can accommodate multiple, individualized goals, and can evaluate and compare change in an individual or group over a heterogenous set of areas [76]. The criteria for success of an intervention thus becomes the extent to which individual goals are achieved rather than the achievement of uniform criteria that are assessed for all patients receiving an intervention [77]. This approach is similar to a disease-specific quality of life measure that allows individuals to choose the disease-specific criteria to be measured and allows individuals to describe their life in ways they consider important [78–80].

One of the main limitations of the GAS score is that if scores are too high then this could mean that the GAS scales developed were not challenging enough or if scores are too low then the scales may be too challenging. The proposed study overcomes this limitation by employing randomization to compare GAS scores between intervention and control groups and uses the same process of asking the patient to self-report changes from baseline to follow-up in both groups so as to minimize measurement bias.

The proposed study is intentional in its interest in being a pragmatic trial. Pragmatic trials ask about effectiveness in a real-world, unrestricted setting of study. They do not ask about efficacy as in explanatory trials that are carried out under selected, often ideal circumstances [81]. Table 4 describes the Health TAPESTRY older adult RCT assessed according to the PRECIS domains [82]. The majority of PRECIS elements are highly pragmatic. One key element following a pragmatic approach is to intentionally allow the interprofessional team to adapt and evolve over time to the participant and volunteer information coming to them compared to setting a fixed rigid process of care mapping to handle the information. This approach specifically aligns with the recognition and acceptance that in the real world the healthcare system epitomizes a complex system that is driven by features such as nonlinearity, emergence, adaptation, uncertainty, dynamical system change, and co-evolution with the need for clinic teams to continuously refine or change their approach over time based on context. It is important in pragmatic trials to also focus on factors influencing implementation of the intervention to support spread of effective interventions to other jurisdictions. Implementation evaluation will provide a rich understanding of the clinic processes so as to help interpret the comparative evaluation results with an understanding of how the implementation unfolds.

**Table 4:** Evaluation of the Health TAPESTRY in older adult RCT assessed according to the PRECIS domains

PRECIS domain	Element	Assessment of the Health TAPESTRY older adult RCT	Research team rating on scale 1 to 5 (1 = least pragmatic to 5 = most pragmatic)
Participants	Participant eligibility criteria	Sample is quite healthy—intentionally left it open and not too targeted at people who are frail; included individuals along bus routes had to screen-out those people who volunteers could not physically access (i.e., long-term care facilities, hospices, along rural routes); had to screen-out individuals who could not speak English or did not have a caregiver who spoke English and willing to facilitate volunteer visits—this was outside the volunteer program’s capacity	4
Interventions and expertise	Experimental intervention-flexibility	TAP-reports reviewed and action plan is developed for that particular person based on their individual self-report information so action plans are all different. There is not a “one size fits all” approach to reports. No specific instructionsgiven to intake teams on process, allowed teams to develop own process and workflow for TAPESTRY; practice was allowed to be different between the two clinics. Never forced clients to use PHR to facilitate connection to healthcare team. At least one home visit by the volunteer was initiated, but no minimum or maximum follow-up visits were enforced in the trial	5
	Experimental intervention-practitioner expertise	MFHT and intake teams made up of different people, intake teams changed throughout the trial, elements of care plan carried out by various MFHT members, regardless of level of expertise	5
	Comparison intervention-flexibility	Control individuals allowed to receive any type of care from any healthcare professional at the clinic. Individuals in the control group do not receive volunteer visits, the TAP-App and are not discussed at the intake team meetings	5
	Comparison intervention-practitioner expertise	Same MFHT members are involved in the care of individuals in the intervention and control groups	5
Follow-up and outcomes	Follow-up intensity	Formal follow-up to collect research outcomes (baseline, 6 and 12 months). No formal volunteer home visit follow-up schedule of clients, no instruction for MFHT of clinic follow-up	4
	Primary trial outcome	Primary out is GAS score, which is subjective, person-centered care outcome and clinically meaningful to client	5
Compliance/adherence	Participant compliance with “prescribed” intervention	No formal strategy to encourage compliance; methods to encourage use of PHR, improve volunteer confidence in role	5
	Practitioner adherence to study protocol	Only subtle strategies to encourage intervention to move forward have left for organic process, but no formal strategy to monitor adherence or encourage adherence; researchers sat in intake team meetings early on to track development of processes, however, have withdrawn researchers have intake teams to lessen influence of initiative	4
	Analysis of primary outcome	Using intention to treat principle, intention to include all experiences in evaluation (good and bad), statistical analysis plan will work under “noise”	5

The pragmatic-explanatory continuum indicator summary (PRECIS) was developed by an international group of interested trialists at two meetings in Toronto (2005 and 2008) and in the time between. The initiative grew from the Pragmatic Randomized Controlled Trials in Health Care (Practihc) project (www.practihc.org), an initiative funded by Canada and the European Union to promote pragmatic trials in low- and middle-income countries [81]. The PRECIS elements that are relatively less pragmatic include the eligibility criteria of participants, follow-up intensity, and practitioner adherence to study protocol, although ratings still represent a highly pragmatic trial. Program reasons for logistical purposes (i.e., excluding people living in areas not easily accessible by bus) and for the collection of process and outcome research measures (i.e., strictly timed follow-up measurement of research outcomes and researchers recording field notes in intake team meetings) have contributed to the lower pragmatic ratings of these elements

The proposed study takes place within a family health team, which may limit its generalizability to other types of primary care settings. Health TAPESTRY has considered the challenge of applicability to other settings during development and has taken steps to assess the sustainability of the integrated care delivery platform and individual components. Formal assessment of sustainability considerations based on the NHS sustainability model [64] has led to decisions and modifications of the intervention during pilot testing that better encourage sustainability and adaptability to other settings [32].

The proposed study has some notable limitations. The evaluation of the Health TAPESTRY platform is in a context with an established climate of collaboration among family healthcare team members with an open source PHR (KindredPHR) linked with an open source electronic medical chart (OSCAR) to facilitate care coordination thereby limiting the generalizability of the results to other clinical settings delivering primary care. However, the open source nature of the e-health ecosystem affords a higher level of opportunity for adoption by other communities. This study will evaluate the way in which elements of Health TAPESTRY can push those processes beyond their current state. Further, the choice to use the outcomes developed by the Canadian Institute of Health Information through the Canadian Institutes of Health Research Community-Based Primary Healthcare initiative [59] to assess the quality of primary care compared to other valid and reliable surveys [83] may limit the findings. Findings from this study may contribute to the evaluation of the psychometric properties of these untested common indicators.

Strengths of our proposed study include its design as a pragmatic RCT, the use of measures that help to understand the effects of the approach from multiple perspectives, the inclusion of a health economics component, and the use of mixed-methods to evaluate the approach. In addition, process and evaluation outcomes are incorporated. In doing so, a complete understanding of all the moving parts of Health TAPESTRY will be captured. Further, the study will engage all key players in the process of implementation, including the clients, volunteers, healthcare team, and researchers involved in the evaluation. Having each perspective will add to the richness of the data and experience throughout the study.

## Additional files

Additional file 1: Secondary research questions and questions related to the process of implementation of each component of the Health TAPESTRY approach. (DOCX 13 kb)

Additional file 2: Study flow and planned timeline. (DOC 69 kb)

Additional file 3: Detailed description of the intervention [57, 84–89]. (DOC 63 kb)

Additional file 4: Screenshot of virtual learning center login page and training modules. (DOC 439 kb)

Additional file 5: The process used to generate a Goal Attainment Scaling (GAS) Score [53, 90]. (DOC 62 kb)

Additional file 6: Cost-effectiveness plan. (DOCX 12 kb)
