# Supplementary material for: A protocol for a pragmatic randomized controlled trial using the Health Teams Advancing Patient Experience: Strengthening Quality (Health TAPESTRY) platform approach to promote person-focused primary healthcare for older adults
Source: Implement Sci. 2016 Apr 5;11:49. doi: 10.1186/s13012-016-0407-5 (PMC4820854; doi:10.1186/s13012-016-0407-5)
Supplement: Supplementary file 1 — Secondary research questions and questions related to the process of implementation of each component of the Health TAPESTRY approach. (DOCX 13 kb) [file 13012_2016_407_MOESM1_ESM.docx]

Additional file 1: Secondary research questions and questions related to the process of implementation of each component of the Health TAPESTRY approach

Four secondary research questions will also be examined, and include the following:

1. What is the effect of the Health TAPESTRY approach on self-efficacy for managing chronic disease, quality of life, optimal aging, social functioning, physical activity, perception of access to primary care services, comprehensiveness, patient empowerment, patient centredness, caregiver stress, satisfaction with health care received at their clinic, and hospitalizations and emergency room visits in older adult participants compared to people not receiving the Health TAPESTRY approach?

2. What is the cost-effectiveness of the Health TAPESTRY approach in older adult participants compared to people not receiving the Health TAPESTRY approach?

3. Are there differences in the effect of the Health TAPESTRY approach depending on age (age less than 80 years compared to 80+ years), sex, those living alone or with others, or those with 3 or more chronic conditions?

4. What is the effect of the Health TAPESTRY approach (on all outcomes) in those who receive the intervention extended for 12 months compared with those who receive it for 6 months? How confident do volunteers feel to fulfill their role?

Research questions related to the process of implementation

1. How complete are the tools intended for administration by the volunteer during in home initial assessments?
2. How satisfied are participants with the volunteer visits?
3. What is the uptake of PHRs by the participants and volunteers?
4. What is the type (communication method, recommendations made) and extent of involvement of the health care team?
5. What is the quality and extent of interdisciplinary health care team functioning and the organizational readiness for change by the health team members?
6. How often and what types of community resources are utilized by participants?
7. What is the type and extent of involvement that participants have in this program?
8. What is the type and extent of involvement of family caregivers in the program?
9. What life, health and care goals are generated by participants?
10. What was the process of implementation and what factors influenced implementation of the intervention including factors to consider when adapting the Health TAPESTRY approach to other contexts?
