# Supplementary material for: A protocol for a pragmatic randomized controlled trial using the Health Teams Advancing Patient Experience: Strengthening Quality (Health TAPESTRY) platform approach to promote person-focused primary healthcare for older adults
Source: Implement Sci. 2016 Apr 5;11:49. doi: 10.1186/s13012-016-0407-5 (PMC4820854; doi:10.1186/s13012-016-0407-5)
Supplement: Supplementary file 2 — Study flow and planned timeline. (DOC 69 kb) [file 13012_2016_407_MOESM2_ESM.doc]

Additional file 2: Study flow (above) and planned timeline (below)

| Recruitment and training of volunteers | | |
| --- | --- | --- |
| Recruitment of participants | | |
| Randomization  C | | |
|  | Intervention | Control |
| Baseline data collection | D | D |
| Intervention | E |  |
| *2 weeks* |  |  |
| *3 weeks* |  |  |
| *1-6 months* |  |  |
| 6-month data collection | J | I |
| *6-12 months* |  | E |
| 12-month data collection | K | K |

|  | 2-3 months (Oct-Dec, 2014) |
| --- | --- |
|  | Rolling fashion, 6-7 months (Dec-June, 2015) |
| C | Randomization once eligibility confirmed and consent signed, 6-7 months (Jan-July, 2015) |
| D | Baseline data collection, rolling fashion 6-8 months (Feb-Sept, 2015) |
|  | Volunteers complete home visits to complete modules on TAP-App for intervention arm, introduce PHR, rolling fashion (Feb-Oct, 2015) |
|  | Health TAPESTRY-report generated and uploaded into EMR (Feb-Oct, 2015) |
|  | Health TAPESTRY-report reviewed at clinic intake meeting by healthcare team, action plan developed and executed (Feb-Nov, 2015) |
|  | Clinic action based on Health TAPESTRY report and intake team suggestions |
|  | Volunteer visits as requested |
| J | 6-month data collection, rolling fashion 8-9 months (Aug-Apr, 2015)  (control participants enter intervention as described above) |
| K | 12-month data collection, rolling fashion 8-9 months (Feb-Oct 2016) |
