# Supplementary material for: A protocol for a pragmatic randomized controlled trial using the Health Teams Advancing Patient Experience: Strengthening Quality (Health TAPESTRY) platform approach to promote person-focused primary healthcare for older adults
Source: Implement Sci. 2016 Apr 5;11:49. doi: 10.1186/s13012-016-0407-5 (PMC4820854; doi:10.1186/s13012-016-0407-5)
Supplement: Supplementary file 3 — Detailed description of the intervention [57, 84–89]. (DOC 63 kb) [file 13012_2016_407_MOESM3_ESM.doc]

Additional file 3: Detailed description of the intervention

A community organization with experience with volunteer management will act as a local lead health TAPESTRY community organization and work with the study team to recruit, train, manage and maintain the volunteers. In addition to an in-person training session, volunteers will complete a set of online training modules through a specially designed virtual learning centre (see Additional File 4 for a visual picture of the virtual learning centre login page and modules) that includes videos, resources, self-assessment quizzes, a learning portfolio, and synchronous communication with others.

Once trained, volunteer pairs will visit clients in their home to collect information electronically using a tablet computer about the client’s health and well-being. The volunteer pair will consist of one person with experience volunteering (usually older) and one university student (usually younger) volunteer. The tablet computer that will be used houses a specifically designed Health TAPESTRY software application that will be used to electronically gather data about life and health goals, daily life activities, general health including risk of frailty, nutrition, memory, advanced directives, mobility, and physical activity using structured surveys and unstructured narratives. Each module is completed by the client, facilitated by the volunteer pair, during a home visit. The volunteer pair will complete as much as possible during the initial visit but continue to visit the client until all the surveys are complete. Information in each module is summarized on a TAPESTRY-report, which is uploaded to the electronic medical record for that client. Information that is critical for review is noted as an “alert”. Alerts are defined in each module below.

1. Daily life activities: questions to understand participants’ daily activities.
2. General health: questions to understand participants’ general overall health and well-being. Included in this APP is the Edmonton Frail Scale (EFS) . A score on the EFS 0-4 is “robust”, 5-6 is “apparently vulnerable” and 7-17 is “frail”.
3. Nutrition: the Screen II [90, 91] will be used and includes questions to understand participants’ eating routine and nutrition. A maximum score is 64 with < 50 being identified as “high risk”.
4. Memory: questions to understand participants’ memory.
5. Advance directives: questions to understand if participants have a set of written instructions which clearly outline what type of medical care participants’ would like in various situations.
6. Mobility: The MANTY is a three-item questionnaire, with several sub-items, administered to inquire about difficulties walking certain distances and climbing stairs. The responses received were categorized as “no difficulty”, “preclinical manifestation”, “minor manifestation” and “major manifestation” with later 3 categories denoting mobility risk based on the literature.
7. Physical activity: the rapid assessment of physical activity (RAPA) is a nine-item scale having two major sections: 1) aerobic activities 2) strength and flexibility. The RAPA is used to assess physical activity level in patients. Scores on the aerobic scale less than 6 indicate sub-optimal activity.
8. Social life: the 10-item duke social support index has two subscales: social network (4 items) and social satisfaction (6 items). A score of less than 10 on the satisfaction sub-scale was identified as “high risk”.
9. Quality of life: the EQ5D-5L has 5 questions with 5 answer options related to mobility, self-care, usual activities, pain/discomfort, anxiety/depression, in addition to a question in which participants rate how good or how bad their health is currently from 0 (worse health imaginable) to 100 (best health imaginable).

Volunteers will also be encouraged to use the activity log, observer notes, and narrative features of the TAP-App. These features are used to track and monitor volunteers time spent in transit to and from visits/ picking up visit materials from the volunteer coordinator, record any additional information deemed important that was uncovered at the home visit, and a subjective description of their home visit and interaction with the client, respectively.

Upon completion of all modules, information is summarized on a TAPESTRY-report, which is uploaded to the electronic medical record by a researcher for that client. Information that is critical for review is noted as an “alert” on the report. Alerts have been defined in each module above. TAPESTRY-reports are then viewed by the intake teams, in addition to the most responsible physician, at each clinic regularly. Upon review, a care plan is developed for clients. The care plan can involve any number of actions, drawing on clinic and community supports and health care professionals.

Volunteers return to the client’s home as instructed by the health care team, the volunteer coordinator or at the request of the client. The volunteers will also assist the clients with setting up their PHR in the home. The PHR linked with the MFHT is called the KindredPHR, which allows the client to record personal health information, book appointments with the clinic, receive documents, send secure messages, use health trackers (e.g., blood pressure, weight) to encourage self-management of health, and allow users to share their record with others if they choose including family and care providers at the clinic. The intention is that the volunteers are formally linked with and considered part of the primary health care team and as such become a set of the Health TAPESTRY team’s eyes, ears, and hands in the home. The volunteer coordinator will follow up with both clients and volunteers to ensure these interactions are going well. A 3-month check-in occurs to review client goal and maintain contact with the client.

Fig 2 represents the general steps of the Health TAPESTRY process.
