# Supplementary figures and images for: A protocol for a pragmatic randomized controlled trial using the Health Teams Advancing Patient Experience: Strengthening Quality (Health TAPESTRY) platform approach to promote person-focused primary healthcare for older adults
Source: Implement Sci. 2016 Apr 5;11:49. doi: 10.1186/s13012-016-0407-5 (PMC4820854; doi:10.1186/s13012-016-0407-5)

Additional file 4: Screenshot of virtual learning centre login page and training modules


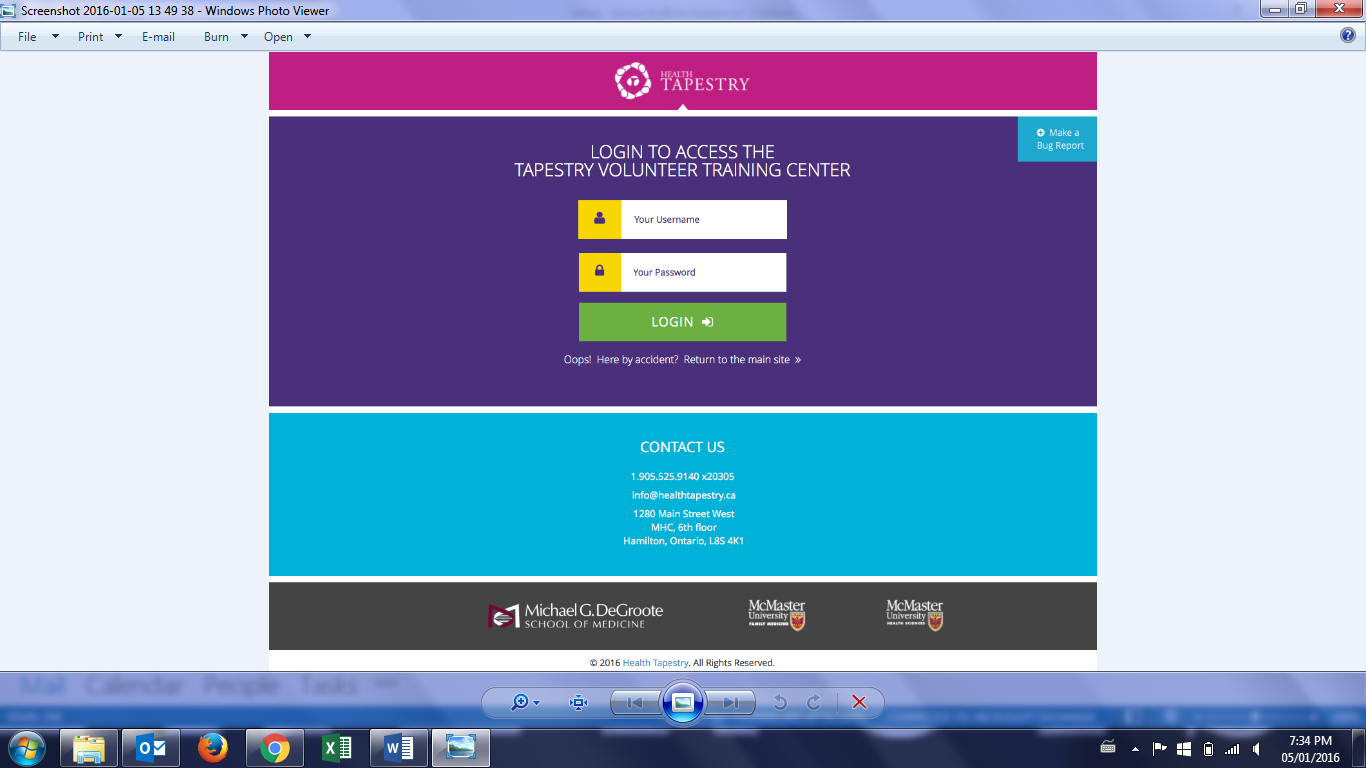


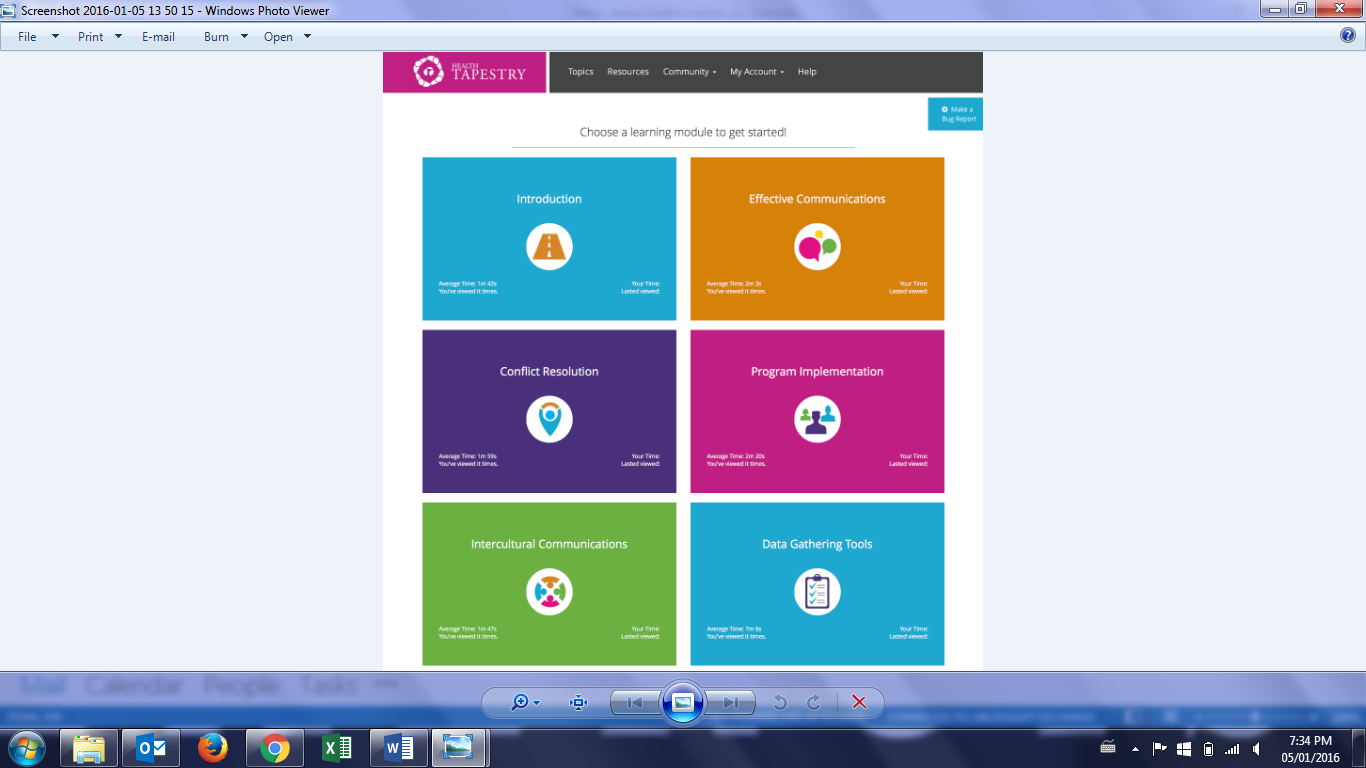

Supplement: Supplementary file 4 — Screenshot of virtual learning center login page and training modules. (DOC 439 kb) [file 13012_2016_407_MOESM4_ESM.doc]
