# Supplementary material for: A protocol for a pragmatic randomized controlled trial using the Health Teams Advancing Patient Experience: Strengthening Quality (Health TAPESTRY) platform approach to promote person-focused primary healthcare for older adults
Source: Implement Sci. 2016 Apr 5;11:49. doi: 10.1186/s13012-016-0407-5 (PMC4820854; doi:10.1186/s13012-016-0407-5)
Supplement: Supplementary file 5 — The process used to generate a Goal Attainment Scaling (GAS) Score [53, 90]. (DOC 62 kb) [file 13012_2016_407_MOESM5_ESM.doc]

Additional file 5: The process used to generate a Goal Attainment Scaling (GAS) Score

Each participant will have a goal setting discussion to identify life and health goal areas using a goal setting script. A goal area is defined as an area of health related to an abnormal sign or laboratory value, bothersome symptom, or clinical or lifestyle problem or prevention area that a participant wants to maintain or change . Up to 3 goal areas related to health (e.g. exercise, nutrition, blood pressure) will be identified and prioritized by the participant. An indicator for each goal will be identified to represent the signs, symptoms, health state, behaviour, skill, or process that can be used to indicate progress (either maintaining or changing) in meeting the goal (e.g. pain, blood pressure, nausea) *.* Specifically, after identifying three goal areas, clients will be asked the following questions:

1. What specifically about [goal area 1] would you like to work on over the next 6 months? The answer to this question will form *specific goal 1 statement*.
2. What are you currently doing about [specific goal 1 statement] or where are you at now with [*specific goal 1 statement*]?
3. What would be ideal, yet possible target for you in achieving [*specific goal 1 statement*]?

These three probe questions will be asked for all three goal areas, forming a “baseline” and 6-month “target” for each of the three goal statements.

A 6-month follow-up goal conversation will occur. Participants are reminded of their 3 specific goal statements and are asked the following questions:

1. Did you reach your goal?
   1. Yes, exceeded expectations
   2. Yes, as expected
   3. Partly achieved
   4. No, same as before
   5. No, worse than before
2. If you reached your goal or partially reached your goal, please explain how
3. If you did not fully reach your goal, please explain why (identifying barriers)
4. What are your next steps with regard to this goal?

A goal attainment scaling score will be calculated for each client using the following equation:

| 50 + | 10S(w_i_x_I_) |
| --- | --- |
|  | √ (1-p)S w_i_^2^ + p(Sw_i_)^2^ |

Where

w_i_ = weight assigned to the _i_th goal

x_i_ = the numerical value (-2 to +2) of the attainment level of the _i_th goal

P= the weighted average intercorrelation of the scale scores (=ICCscale = variance due to the difference among scales / total variance)

The goal attainment scaling formula produces an overall score which is a mean of the outcome scores adjusted for: (a) relative weighting assigned to the goals, (b) varying number of goals, (c) expected inter-correlation among the goal scales.  A score of 50 indicates that all goals are met at the expected level. For example, a score of less than 50 means that a client did not improve as much as expected when all of the medical problems areas assessed by goal attainment scaling were considered.

The goal attainment scaling score measures the degree of change for an individual during the treatment period relative to the amount of change predicted. It is not an absolute measurement of the level of post treatment status. If scores are too high then this could mean that the goal attainment scaling score developed were not challenging enough or if scores are too low then the scales may be too challenging. Because of its properties as a measure of change, the goal attainment scaling score provides a measure of change from baseline and not a measure of post-intervention status. Therefore, the score would be expected to correlate with measures of change or post-intervention minus baseline scores rather than standardized normative treatment measures that only assess post-intervention status. For example, a client could undergo a change that could be considered large for that client but not large relative to others and so the client’s score could still be at the low end of a normative scale.

The indicators and outcomes will be applied to the intervention group and control group in the same manner however the goal areas will be identified during discussion with volunteers in the intervention group and by the research staff in the control group. Pilot testing of the goal setting process provided an opportunity to refine the processes of asking participants probing questions so as to generate enough (at least three) priority goal areas to work toward. The goal attainment scaling score will be used as the primary outcome. Also, the proportion of participants self-reporting maintenance or improvement in their top priority goal area will be used as a secondary outcome.
