# Supplementary material for: A protocol for a pragmatic randomized controlled trial using the Health Teams Advancing Patient Experience: Strengthening Quality (Health TAPESTRY) platform approach to promote person-focused primary healthcare for older adults
Source: Implement Sci. 2016 Apr 5;11:49. doi: 10.1186/s13012-016-0407-5 (PMC4820854; doi:10.1186/s13012-016-0407-5)
Supplement: Supplementary file 6 — Cost-effectiveness plan. (DOCX 12 kb) [file 13012_2016_407_MOESM6_ESM.docx]

Additional file 6: Cost-effectiveness plan

Program costs will be measured using micro costing techniques using a standardized and then pilot tested health care cost and resource utilization form. This resource use information will be combined with unit prices for each resource and summed across all resources to estimate the total cost of the program. Costs to carry out the research evaluation will not be included. One-time development costs for Program infrastructure (e.g. the Virtual Learning Centre, the PHR) will be calculated but not be included since these costs are nonrecurring. Maintaining and updating infrastructure costs will be included. Health care resource use and costs will be obtained from the participant questionnaire that will assess cost and resources involved in providing health care for older adults. Questions regarding number of visits to emergency, family doctor, specialist; number of hospital admissions; use of professional care services, home care services, supporting equipment and medications; number of care givers and the hours devoted per week by them are included in the questionnaire. The EMR will be used to confirm self-report data of hospitalizations, emergency room visits, urgent care visits, and prescription drug use. Utilities will be measured using the EQ-5D-5L.
